# Draft genome sequencing of human pathogenic *Vibrio fluvialis* BD001 strain isolated from shrimp (*Penaeus monodon*) in Bangladesh

**DOI:** 10.1128/mra.00535-25

**Published:** 2025-07-07

**Authors:** Md. Iftehimul, Sakib Anzum Pranto, Mishkatul Jannat Megha, David Bass, Muhammad Tofazzal Hossain, Mohammad Mahfujul Haque, Morena Santi, Neaz A. Hasan

**Affiliations:** 1 Department of Biotechnology, Bangladesh Agricultural University 54492, Mymensingh, Bangladesh; 2 Department of Microbiology and Hygiene, Bangladesh Agricultural University 54492, Mymensingh, Bangladesh; 3 Holy Family Red Crescent Medical College 469106, Dhaka, Bangladesh; 4 International Centre of Excellence for Aquatic Animal Health, Centre for Environment, Fisheries and Aquaculture Science, Weymouth Laboratory, Dorset, United Kingdom; 5 Department of Aquaculture, Bangladesh Agricultural University 54492, Mymensingh, Bangladesh; 6 Department of Fisheries and Marine Bioscience, Gopalganj Science and Technology University 823103, Gopalganj, Bangladesh; Montana State University, Bozeman, Montana, USA

**Keywords:** *Vibrio fluvialis*, shrimp, *Penaeus monodon*

## Abstract

We report the isolation and genome sequence of *Vibrio fluvialis* BD001 from shrimp in Bangladesh. The assembled genome of BD001 strain is ~4.8 Mbp in size, with 50% GC content and 99× coverage depth. The genome reveals insights into antibiotic resistance, virulence, and potential human pathogenicity.

## ANNOUNCEMENT

*Vibrio fluvialis* is a water- or seafood-borne pathogen that causes gastroenteritis and extraintestinal infection, with the potential to become endemic in Bangladesh and pose a serious public health risk (1). This report presents the first known isolation of *V. fluvialis* from shrimp (*Penaeus monodon*) samples in Khulna, Bangladesh, emphasizing its possible emergence as a pathogen of concern for food safety and aquaculture. *P. monodon*, the country’s main farmed shrimp species (2–5), is largely export-oriented and supports the seafood supply chain, economic growth, and foreign exchange with major export destinations to European countries, including Italy, the United Kingdom, Germany, the Netherlands, Portugal, and Belgium (6, 7).

To isolate BD001, intestinal samples were collected from shrimp and transferred to Brain Heart Infusion (BHI, Oxoid, UK) broth with 2% NaCl (FUJIFILM Wako Pure Chemical Corporation, Japan). After overnight bacterial enrichment at 37°C, samples were streaked multiple times onto thiosulfate-citrate-bile salts-sucrose agar (Oxoid, UK) to obtain pure colonies (8). Afterward, BD001 was phenotypically characterized by yellowish, round-shaped, and gram-negative staining (9). For genomic DNA extraction, a single colony from the original stock was cultured in BHI broth (2% salt) at 37°C for 10 h (10), followed by DNA isolation using the GeneJET Genomic DNA Purification Kit (Thermo Fisher Scientific, USA), according to the manufacturer’s instructions. DNA purity and quantity were measured by Nanodrop (Thermo Fisher Scientific, USA). DNA library was prepared using the Nextera XT Library Prep Kit (Illumina, San Diego, CA, USA), and sequencing was performed on the Illumina MiSeq platform with UDPO101 adapters, where strain BD001 produced 1,012,822 paired-end spots and 471.1 million bases. Read quality was assessed using FastQC v.0.12.1 (11), and low-quality sequences were trimmed using Fastp v. 0.24.0 (12). High-quality reads were assembled into *de novo* genomes using SPAdes v.4.0.0 (13), and QUAST v.5.3.0 evaluated the assembly quality (14). Annotation was performed with NCBI Prokaryotic Genome Annotation Pipeline (15) and CheckM version 1.2.3 assessed completeness and contamination (16). Antibiotic resistance genes (ARGs), plasmids, and virulence factor genes were identified using ResFinder, PlasmidFinder, and VFDB, respectively, within the ABRicate tool on the Linux command line (17–19). RAST v2.0 web server was used to identify subsystems (20), and PathogenFinder v1.1 evaluated pathogenicity (21). BAGEL4 (22), gutSMASH v1.0 (23), and antiSMASH v7.0 (24) were used to predict bacteriocins, primary metabolic, and secondary metabolic gene clusters, respectively. PHASTEST and ICEFinder identified prophages and CRISPR arrays (25, 26). All genomic analyses were performed using default parameters.

The assembled genome (~4.8 Mbp) consists of 106 contigs, the largest being 376,976 bp, with 50% GC content, N50 of 248.5 kbp, and L50 of 8. Sequencing depth reached 99× coverage, and the highest Average Nucleotide Identity (ANI) was observed with the reference genome (GCF_001558415.2) with an ANI value of 97.22% as calculated using EzBioCloud (27). Genome completeness was 99.54%, with 1.14% contamination. Annotation identified several virulence-associated genes (*vipB/mglB*, *hcp-2*, and *luxS*) and ARGs (*tetB* and *blaTEM-116*). The genome contains 4,522 genes: 4,343 protein-coding and 121 RNA genes (99 tRNAs, 18 rRNAs (5 complete 5S rRNAs), and 4 ncRNAs), indicating a well-equipped translation system. RAST identified 370 metabolic subsystems; gutSMASH and antiSMASH detected 19 primary and 7 secondary metabolite gene clusters. PathogenFinder v1.1 predicted BD001 as a potential human pathogen (score 0.661) with 0.75% proteome coverage and 24 pathogenic and 9 non-pathogenic protein matches. No plasmid, CRISPR array, prophages, or bacteriocins were detected.

## Data Availability

The whole genome sequence of *Vibrio fluvialis* has been deposited at GenBank under the accession number JBMIMY000000000. The associated BioProject and BioSample accession numbers are PRJNA1234149 and SAMN47579942, respectively. The raw data are available from the Sequence Read Archive (SRA) under accession number SRR32897651.
